# Prevalence of incorrect body posture in children and adolescents with overweight and obesity

**DOI:** 10.1007/s00431-017-2873-4

**Published:** 2017-02-22

**Authors:** Katarzyna Maciałczyk-Paprocka, Barbara Stawińska-Witoszyńska, Tomasz Kotwicki, Anna Sowińska, Alicja Krzyżaniak, Jarosław Walkowiak, Małgorzata Krzywińska-Wiewiorowska

**Affiliations:** 1 Physical Education and Sports Department, Poznań University of Medical Sciences, Marcelińska 25, 60-802 Poznań, Poland; 2 Department of Epidemiology Chair of Social Medicine, Poznań University of Medical Sciences, Dąbrowskiego 79, 60-529 Poznań, Poland; 3 Department of Pediatric Orthopedics and Traumatology, Poznań University of Medical Sciences, Poznań, Poland; 4 Department of Computer Science and Medical Statistics, Poznań University of Medical Sciences, Poznań, Poland; 5 Department of Pediatric Gastroenterology and Metabolic Diseases, 1st Chair of Pediatrics, Poznań University of Medical Sciences, Poznań, Poland

**Keywords:** Prevalence, Posture, Overweight, Obesity, Child, Adolescents

## Abstract

The ever increasing epidemics of overweight and obesity in school children may be one of the reasons of the growing numbers of children with incorrect body posture. The purpose of the study was the assessment of the prevalence of incorrect body posture in children and adolescents with overweight and obesity in Poznań, Poland. The population subject to study consisted of 2732 boys and girls aged 3–18 with obesity, overweight, and standard body mass. The assessment of body mass was performed based on BMI, adopting Cole’s cutoff values. The evaluation of body posture was performed according to the postural error chart based on criteria complied by professor Dega. The prevalence rates of postural errors were significantly higher among children and adolescents with overweight and obesity than among the group with standard body mass. In the overweight group, it amounted to 69.2% and in the obese group to 78.6%.

*Conclusion:* The most common postural deviations in obese children and adolescents were valgus knees and flat feet. Overweight and obesity in children and adolescents, predisposing to higher incidence of some types of postural errors, call for prevention programs addressing both health problems.

**Table:** 

**What is Known:** • *The increase in the prevalence of overweight and obesity among children and adolescents has drawn attention to additional health complications which may occur in this population such as occurrence of incorrect body posture*.
**What is New:** • *The modified chart of postural errors proved to be an effective tool in the assessment of incorrect body posture*. • *This chart may be used in the assessment of posture during screening tests and prevention actions at school*.

## Introduction

Body posture failures in children and adolescents constitute one of the most popular yet underestimated health problems. The reduction in cardio-respiratory efficiency, decreased vital capacity of the lungs, degenerative bone and low back pains, as well as the displacement of the internal organs are just some of the consequences of untreated incorrect body posture [46].

According to the data published by *Haslam* and *James*, about 10% of the world population aged up to 18 are overweight or obese, and American studies of children and adolescents indicate excessive body mass in about 30% of the subjects [13, 14, 43]. In Europe, there are about 20% children with excessive body mass, 5% of whom suffer from obesity [27, 28].

The increase in the prevalence of overweight and obesity among children and adolescents has drawn attention to additional health complications which may occur in this population. Most of the studies concern cardiovascular complications. Subclinical organ damage seen in children with obesity, manifested in hypertension, left ventricular hypertrophy, and dysfunction, as well as thickening of intima-media carotid arteries, can all cause worse cardiovascular adaptation to physical effort and faster fatigue, resulting in the decline in physical fitness [4, 9, 38, 40]. In adults, degenerative diseases of the musculoskeletal system, osteoporosis, and low back pain syndromes are more and more often quoted among the complications related to obesity [6, 24, 34]. There are very few studies devoted to the prevalence of incorrect body posture in children and adolescents with excessive body mass [25, 32]. Low physical activity accompanying obesity may also cause the emergence of constrictions. Sedentary lifestyle constitutes an additional factor not only in increasing the risk of overweight and obesity, but also when it comes to intensifying the prevalence of incorrect body posture in school children. Even though Poland is one of the countries where adolescents are exposed to the recommended level of physical activity, the number of children with poor body posture is still high [8, 33]. The assessment of body posture should constitute a crucial element of the complex examination of children and adolescents with excessive body mass. Considering the large number of parameters subject to assessment, this method should be simple.

There are numerous definitions of body posture and its assessment methods [7, 21, 44]. The reference posture could be defined by the relationship between the gravity line and the body segments. [47]. According to prof. Dega, the definition of body posture should incorporate not only the vertical alignment of the torso but also shoulders, lower limbs, and feet, as well as the shape of abdominal walls [7]. Kasperczyk defines body posture as the setting of particular body sections unaffected by pathological changes, providing optimal body stability, requiring minimum muscular effort, thus creating optimal conditions for the positioning of internal organs [21].

The ever increasing epidemics of overweight and obesity, as well as the growing prevalence of incorrect body posture in children and adolescents, call for some actions which would lead to an increase in physical activity and draw attention to appropriate dietary habits. These activities constitute an important element of prevention of osteoporosis, osteoarthritis, pain in the sacral region of the spine, and the elimination of other organ complications related to obesity [12, 45].

## Purpose

The purpose of the study was the epidemiological assessment of the prevalence of incorrect body posture in children and adolescents with overweight and obesity resident in Poznań, Poland.

## Materials and methods

The study population was a representative group of children and adolescents aged 3–18, randomly chosen from kindergartens (3–6), primary schools (7–12), and junior and secondary schools (13–18) of Poznań, Poland.

The duration of the study was 2 years. Study population was selected randomly, and the sampling frame encompassed kindergartens, primary, junior, and secondary schools in particular residential districts. Examination included 1363 boys and 1369 girls, which constituted 3.5% of the overall population of children and adolescents aged 3–18 living in Poznań. All parents and their children gave their informed consent prior to their inclusion in the study.

For the assessment of postural errors, only those children were qualified in whom, based on an overall pediatric examination, birth defects, trauma, and chronic bone and joint diseases that could affect the body statics disorders had been excluded.

The measurements of body height and mass were performed in accordance with the principles of anthropometrics, and the calculations of correct body mass, overweight, and obesity were based on the adopted body mass index (BMI) according to the formula BMI = body mass expressed in kg/(body height)^2^ expressed in meters, adopting the cutoff values of standard weight, overweight, and obesity according to Cole’s criteria [3, 44]. BMI value 90 ≤ 95th percentile was qualified as overweight; BMI value >95th percentile was considered obesity. The studied population is presented with respect to BMI category and gender in Table 1.

**Table 1:** Studied population by BMI, age, and gender

Age (years)	3–6			7–12			13–18			Total		
Sex	Boys	Girls	*p* value	Boys	Girls	*p* value	Boy	Girls	*p* value	Boys	Girls	*p* value
BMI categories	*n* (%)	*n* (%)		*n* (%)	*n* (%)		*n* (%)	*n* (%)		*n* (%)	*n* (%)	
Norm	203 (87.1)	192 (85.3)	0.590^a^	444 (84.7)	444 (85.5)	0.728^a^	522 (86.1)	550 (88.0)	0.350^a^	1169 (85.8)	1186 (86.6)	0.542^a^
Overweight	18(7.7)	23(10.2)		41(7.8)	28(5.3)		39(7.4)	36(5.8)		98(7.2)	67(6.3)	
Obesity	12(5.1)	10(4.4)		39(7.4)	47(9.0)		45(7.5)	39(6.2)		96(7.0)	96(7.0)	

^a^Chi-square test

*p* ≤ 0.05 level of significance

The assessment of prevalence of incorrect body posture in the group of children with normal weight and in the group with overweight and obesity (excessive body mass) was performed according to the postural error chart based on criteria complied by professor Dega [7]. The examination was performed by rehabilitation expert. The postural error chart comprised 15 body posture characteristics, examined according to the visual assessment method, including appropriate functional tests to check the muscular constrictions (elasticity tests of pectorals, ilio-lumbar, and ischiocrural muscles were performed) (Table 2) [7].

Every child in underwear was analyzed with respect to the occurrence of particular postural errors in three planes (frontal, diagonal, and sagittal). The assessment encompassed the alignment of head, neck, shoulders and shoulder blades, chest shape, formation of spinal curvatures—the course of lordosis and kyphosis. The shape of abdominal walls, pelvis, and lower limbs was also analyzed. During the examination of the spine run, the child standing with his/her back to the examiner was told to loosely bend forward (feet slightly apart, head and arms hanging down loose). The examiner, having conducted the index and middle fingers along the spinous processes, could then tell (after the child had returned to the erect position) if the student had scoliotic posture or not.

According to the recommendations of the method author, the occurrence of muscular contractures was assessed, based on specific functional tests: Thomas’ test (examining the presence of iliolumbar muscles), the tests evaluating the contractures of ischiocrural muscles, as well as the test indicating the presence of contracture in shoulder joints [7, 21]. The flexibility of iliolumbar, ischiocrural, and shoulder muscles was diagnosed in both limbs.

The presence of a postural error related to specific sections of the body was marked on the chart with digit “1,” and if no deviations were observed, digit “0” was entered. If the studied subject had at least one postural error, he/she was qualified into the group with incorrect body posture. The postural error chart is presented in Table 2.

**Table 2:** Specification of postural errors by Dega’s criteria

Table of postural errors																	
Head	Shoulders	Pectus carinatum	Pectus excavatum	Hyperkyphosis	Scoliotic posture	Scoliosis	Hyperlordosis	Pelvis	Abdomen	Flat back	Genu varum	Genu valgum	Flat feet	Valgus foot	Muscular constrictions		
															Shoulder joint	Hip-joint	Knee-joint

Statistical calculations were performed by means of *Statistica 10.0* and *Cytel Studio 10.0 software.* The significance of differences between the postural error incidence rates expressed in terms of gender, age, and BMI was calculated based on a difference test, assuming the level of *p* < 0.05 for the significant values. The assessment of postural error incidence rates depending on BMI was checked by means of Chi-square test, Fisher’s exact test, and Chi-square test with Yates’ correction.

The postural error occurrence odds in terms of BMI was calculated on the basis of odds ratio (OR) with 95% confidence interval (CI).

## Results

In the whole population included in the study (2732 subjects), 67.9% of the examined subjects (918 boys and 938 girls) had at least one postural defect.

In obese children, the prevalence of postural defects was significantly higher than among the children with normal body mass (*p* = 0.001) and overweight children (*p* = 0.0046) (Table 3).

**Table 3:** Errors in body posture in the whole population by BMI category

	Error present *n* (%)	No error *n* (%)	*p* value^a^
Norm	1577 (67.0)	778 (33.0)	0.570^b^
Overweight	128 (69.2)	57 (30.8)	0.001^c^
Obesity	151 (78.6)	41 (21.4)	0.046^d^

^a^Chi-square test

^b^Norm vs overweight

^c^Overweight vs obesity

^d^Obesity vs norm

*p* ≤ 0.05 level of significance

Among girls and boys with excessive body mass (overweight and obesity), the percentage of postural errors was the highest in the 7–12 age group (Table 4). Despite the fact that in girls with excessive body mass aged 7–12 as well as 13–18, the number of postural errors was higher than that in boys, the difference was not statistically significant (*p* > 0.05).

**Table 4:** The prevalence of postural errors with contractures in both sexes and total population in terms of BMI categories and age

Sex	BMI category	Age-years			Postural errors		Total
		3–6	7–12	13–18	Total errors present	Total no. of errors	
		*n* (%)	*n* (%)	*n* (%)	*n* (%)	*n* (%)	*n*
Boys	Norm	147 (72.4)	303 (68.2)	333 (63.8)	783 (66.98)	386 (33.02)	1169
	Overweight	13 (72.2)	32 (78)	17 (43.6)	62 (63.3)	36 (36.7)	98
	Obesity	9 (75)	33 (84.6)	31 (68.9)	73 (76.8)	26 (23.2)	96
	Total	169 (72.5)	368 (70.2)	381 (62.9)	918 (67.3)	445(32.7)	1363
Girls	Norm	126 (65.6)	303 (68.2)	365 (66.4)	794 (66.9)	392 (33.1)	1186
	Overweight	19 (73)	23 (82.1)	24 (66.7)	66 (75.8)	21 (24.2)	87
	Obesity	7 (70)	41 (87.2)	30 (76.9)	78 (81.2)	18 (18.8)	96
	Total	152 (67.5)	367 (70.7)	419 (67)	938 (68.5)	431 (31.5)	1369
Total	Norm	273 (69.1)	606 (68.2)	698 (65.1)	1577 (66.96)	778 (33.04)	2355
	Overweight	32 (74.4)	55 (79.7)	41 (54.7)	128 (69.2)	57 (30.8)	185
	Obesity	16 (72.7)	74 (86)	61 (72.6)	151 (78.6)	41 (21.4)	192
	Total	321 (70.1)	735 (70.5)	800 (64.98)	1856 (67.9)	876 (32.1)	2732

The prevalence rate of incorrect body posture in all obese boys turned out to be 1.5 times higher than among boys with normal body mass. This difference is statistically insignificant. In obese girls, the postural error prevalence ratio was two times higher than in the group with correct body mass and that difference proved to be significant (*p* = 0.004) (Table 5). The prevalence rates of postural errors in overweight and obese children in comparison with the studied subject with standard body mass as divided into specific age groups are presented in Table 6.

**Table 5:** Chance of abnormal posture occurrence in children with overweight and obesity compared to subjects with normal body weight

Boys	IBP	CBP	OR (95% CI)	*p* value
Norm	783	386	Reference	–
Overweight	62	36	0 849 (0.544–1.343)	0.520
Obesity	73	23	1.565 (0.950–2.662)	0.082
Girls				
Norm	794	392	Reference	–
Overweight	66	21	1.552 (0.921–2.710)	0.106
Obesity	78	18	2.139 (1.247–3.850)	0.004

*IBP* incorrect body posture, *CBP* correct body posture, *OR* odds ratio

*p* ≤ 0.05 level of significance

**Table 6:** The chance of postural error occurrence in overweight and obese children and as compared to subjects with normal body weight in different age groups

Age-years	Sex	BMI categories	Errors present	No error	OR (95% CI)	*p* value
3–6	Boys	Norm	147	56	Reference	–
		Overweight	13	5	0.991 (0.313–3.715)	1.000
		Obesity	9	3	1.143 (0.272–6.794)	1.000
	Girls	Norm	126	66	Reference	–
		Overweight	19	4	2.488 (0.779–10.43)	0.151
		Obesity	7	3	1.232 (0.268–7.555)	1.000
7–12	Boys	Norm	303	141	Reference	–
		Overweight	32	9	1.655 (0.746–4.046)	0.259
		Obesity	33	6	2.559 (1.026–7.633)	0.042
	Girls	Norm	303	141	Reference	–
		Overweight	23	5	2.141 (0.773–7.348)	0.175
		Obesity	41	6	3.180 (1.299–9.362)	0.007
13–18	Boys	Norm	333	189	Reference	–
		Overweight	17	22	0.439 (0.213–0.891)	0.021
		Obesity	31	14	1.257 (0.630–2.623)	0.689
	Girls	Norm	365	185	Reference	–
		Overweight	24	12	1.014 (0.475–2.277)	1.000
		Obesity	30	9	1.689 (0.761–4.129)	0.235

*OR* odds ratio

*p* ≤ 0.05 level of significance

**Table 7:** Types of postural errors by BMI category in children aged 3–6 years

Error location	Norm *n* (%)	Overweight *n*	Obesity *n*	Overweight and obesity *n* (%)	*p* value^a^
Head	35 (8.8)	6	1	7 (11.1)	0.637^b^
Shoulders	152 (38.5)	19	8	27 (42.8)	0.578^b^
Pectus carinatum	28 (7.1)	3	0	3 (4.8)	0.679^c^
Pectus excavatum	11 (2.8)	2	2	4 (6.3)	0.274^c^
Hyperkyphosis	31 (7.8)	5	1	6 (9.5)	0.804^b^
Scoliosis	42 (10.6)	4	5	9 (14.3)	0.518^b^
Scoliotic posture	46 (11.6)	4	1	5 (7.9)	0.407^b^
Hyperlordosis	16 (4.0)	2	0	2 (3.2)	0.972^c^
Pelvis	24 (6.1)	4	0	4 (6.3)	0.835^c^
Abdomen	59 (14.9)	9	7	16 (25.4)	0.044^b^
Flat back	0 (0.0)	0	0	0 (0.0)	–
Genu varum	6 (1.5)	0	0	0 (0.0)	1.000^d^
Genu valgum	6 (1.5)	2	1	3 (4.8)	0.217^c^
Flat feet	33 (8.3)	2	2	4 (6.3)	0.762^b^
Valgus foot	2 (0.5)	0	0	0 (0.0)	1.000^d^
*N*	395	41	22	63	

^a^Norm vs overweight and obesity

^b^Chi-square test

^c^Chi-square test with Yates correction

^d^Precise Fisher test

*p* ≤ 0.05 level of significance

**Table 8:** Types of postural errors by BMI category in children aged 7–12 years

Error location	Norm *n* (%)	Overweight *n* (%)	Obesity *n* (%)	Overweight and obesity *n* (%)	*p* value^a^
Head	126 (14.4)	1	3	4 (2.6)	0.0001^b^
Shoulders	260 (29.6)	10	9	19 (12.2)	0.00001^b^
Pectus carinatum	39 (4.4)	1	0	1 (0.6)	0.037^b^
Pectus excavatum	21 (2.4)	1	0	1 (0.6)	0.234^c^
Hyperkyphosis	62 (7.1)	0	3	3 (1.9)	0.017^b^
Scoliosis	80 (9.1)	4	3	7 (4.5)	0.081^b^
Scoliotic posture	116 (13.2)	5	3	8 (5.2)	0.007^b^
Hyperlordosis	58 (6.6)	2	2	4 (2.6)	0.064^b^
Pelvis	51 (5.8)	5	5	10 (6.4)	0.853^b^
Abdomen	59 (6.7)	17	25	42 (27.1)	<0.00001^b^
Flat back	16 (1.8)	0	4	4 (2.6)	0.728^c^
Genu varum	6 (0.7)	1	3	4 (2.6)	0.080^c^
Genu valgum	93 (10.6)	33	56	89 (57.4)	<0.00001^b^
Flat feet	114 (13.0)	17	14	31 (20.0)	0.022^b^
Valgus foot	75 (8.5)	7	11	18 (11.6)	0.221^b^
*N*	888	69	86	155	

^a^Norm vs overweight and obesity

^b^Chi-square test

^c^Chi-square test with Yates correction

*p* ≤ 0.05 level of significance

**Table 9:** Types of postural errors by BMI category in children aged 13–18 years

Error location	Norm *n* (%)	Overweight *n* (%)	Obesity *n* (%)	Overweight and obesity *n* (%)	*p* value^a^
Head	81 (7.5)	3	3	6 (3.7)	0.096^b^
Shoulders	183 (17.1)	9	6	15 (9.4)	0.015^b^
Pectus carinatum	39 (3.6)	3	0	3 (1.9)	0.350^b^
Pectus excavatum	34 (3.2)	0	0	0 (0.0)	0.016^d^
Hyperkyphosis	105 (9.8)	1	0	1 (0.6)	0.00002^b^
Scoliosis	183 (17.1)	12	11	23 (14.5)	0.429^b^
Scoliotic posture	203 (18.9)	14	8	22 (13.8)	0.125^b^
Hyperlordosis	69 (6.4)	1	5	6 (3.7)	0.217^b^
Pelvis	75 (7.0)	2	5	7 (4.4)	0.240^b^
Abdomen	27 (2.5)	1	2	3 (1.9)	0.836^c^
Flat back	22 (2.0)	1	1	2 (1.2)	0.712^c^
Genu varum	99 (9.2)	1	7	8 (5.0)	0.096^b^
Genu valgum	47 (4.4)	14	38	52 (32.7)	<0.00001^b^
Flat feet	42 (3.9)	3	9	12 (7.5)	0.041^b^
Valgus foot	15 (1.4)	0	2	2 (1.2)	0.825^c^
*N*	1072	75	84	159	

^a^Norm vs overweight and obesity

^b^Chi-square test

^c^Chi-square test with Yates correction

^d^Precise Fisher test

*p* ≤ 0.05 level of significance

In children aged 3–6, obesity and overweight have not increased the chances of postural errors.

In the group of students aged 7–12, the probability of occurrence of postural errors was significantly higher among the obese ones than among the students with normal body mass, both in boys (*p* = 0.042) and in girls (*p* = 0.007) (Table 6).

Only in overweight boys aged 13–18, the number of postural errors was significantly lower than that in their peer group with correct body mass (*p* = 0.021) (Table 6).

The most frequently observed postural errors in children with excessive body mass aged 3–6 were incorrect shoulder alignment and protruding abdomen; however, only the latter error was recognized in this age group significantly more often in comparison to the group with normal body mass (*p* = 0.044) (Table 7).

The most frequently observed postural errors in overweight and obese pupils aged 7–12 were valgus knees, incorrect abdominal alignment, and flat feet, and in comparison to the children with standard body mass, those differences proved to be statistically significant (Table 8).

In the 13–18 age group of obese and overweight students, valgus knees (*p* = 0.0001) and flat feet (*p* = 0.041) were observed more frequently than among their peers with normal body mass (Table 9).

Constriction in at least one joint occurred mostly among the examined population with postural errors; 93.5% of the studied subjects with postural errors had at least one constriction. Among the studied subjects with no postural errors, this percentage equalled 6.5 and the difference was statistically significant (*p* < 0.05).

## Discussion

Presented study shows that 74% children with excessive body mass had postural errors. Among kindergartners aged 3–6, there occurs a rapid body height increase and the posture is characterized by a protruding abdomen, which was observed significantly more often in obese children (25.4%) in comparison to children with standard body mass (14.9%). This is probably associated with evident lumbar lordosis and slight thoracic kyphosis. This age group was also characterized by the highest prevalence rates of incorrect shoulder alignment (due to diversified dynamics of bone and muscle growth) and the occurrence of constrictions. With respect to incorrect shoulder alignment, there were no statistically significant differences observed between the group of children with standard body mass and those with excessive body mass.

Another group of postural errors that occurred more frequently in obese teenagers aged 13–18, as compared with the study subjects with normal body mass which may result in serious orthopedic complications, was valgus knees and flat feet. Postural assessment of lower limbs is important for differentiation between physiological and pathological alterations of development. In Brazilian study, a significant difference in the occurrence of knee and ankle pattern posture was found between obese and normal weight children [18]. Recent systematic review confirmed higher percentage of flat foot in obese children [37]. Obese children reveal decreased physical fitness [39]. Excessive joint laxity and obesity increase the tensile strength resulting in development of flexible flat foot. Mueller et al. observed significant midfoot overload during gait in the obese comparing to the normal weight children [30]. The knee axis undergoes changes during growth: there exists physiological varus at infancy while after the age of 4–5 years, the valgus knee of 7–10 degrees is present. Obesity increases the loading of distal femoral and proximal tibial growth cartilages which can result in higher prevalence of the valgus knee.

Epidemiological data related to the prevalence of incorrect body posture in Poland are very diversified [8, 19, 21–23]. This may stem not only from different age of the studied subjects, but also from diversified diagnostic criteria, as well as environmental conditions [19, 22, 23]. The performed tests did not indicate any significant differences in the prevalence of incorrect body posture between boys and girls in specific age groups. The limitations of this study include reliability because of qualitative nature of the method and its dependency on examiner ability. However, this method is not considered a diagnostic tool; it is rather seen as a type of screening test.

The increase of the number of overweight and obese children and adolescents was observed in Poland that can lead to increased prevalence of already widespread phenomenon of incorrect body posture in this population. The occurrence of faulty posture, most often habitual faulty posture, in children and adolescents in Poland, a few years ago was estimated at around 50–60% depending on the region of the country [10].

Most of the studies pertaining to children and adolescents do not take into consideration the body mass criterion. Only the authors of epidemiological studies related to juvenile idiopathic scoliosis underline the higher postural error occurrence risk, especially among slim girls with rapid rate of weight gain [1, 2, 11, 20, 36].

In this study, the prevalence of overweight among boys in three age groups (3–6, 7–12, and 13–18 years) was at a similar level. The percentage of obesity among boys aged 7–12 and 13–18 years (7.4 and 7.5%) was higher than among boys between 3 and 6 years of age. The highest percentage of overweight girls (10.2%) was observed in the youngest age range of 3–6 years, while obese in the age group 7–12 years (9%). There were no significant differences between body weight of boys and girls in these three age groups.

A meta-analysis of 450 cross-sectional studies conducted in different countries on a representative sample of respondents showed an increase in the proportion of preschool children with overweight and obesity from 4.2% in 1990 to 6.7% in 2010. Most children who were diagnosed with overweight or obesity (35 million to 43 million diagnoses) lived in developing countries [5].

At school (i.e., at the age of 7–12), children have mastered all simple motion activities and they start to show interest in sports activities. Consequently, it is a very good time for the formation of increased physical and sports activity habits. At the same time, it is the period in which children’s posture tends to be very sensitive to the changes in environmental conditions. At the age of 7–12, the so far spontaneous and natural physical activity gets limited. This is associated with prolonged periods of being in a seated position during lessons at school and spending leisure time in front of computers and TV screens. In this age group, the abdominal obesity was significantly more often observed among adolescents with excessive body mass. The studies on adults indicated that the waist circumference was the only component that was positively correlated to the mineral density of the bones [15].

Study published by Nawarycz-Ostrowska et al. indicated an increased abdominal circumference among obese children when compared to the children with normal body mass [31].

Consequently, it seems that the assessment of waste/hip ratio or abdominal circumference would be indispensable in these age groups, being a simple measurement that could facilitate continuous monitoring of one of the elements of physical development. The negative impact of increased visceral body fat mass on skeletal system and calcium management is more and more often drawn attention to, mostly in the studies of adults, in which low concentration of vitamin D was observed in obese people [15–17].

Postural failures (incorrect body posture) observed at the age of 13–18 years may be associated with the speed of puberty. It is the period characterized by great dynamics in the increase of body height and fat gain (especially in the breasts, hips, and buttocks). Also, it is when the dimensions of chest and shoulders get larger and a considerable body mass growth is observed. Girls, especially the tall ones, tend to slouch and incorrectly position their shoulders. This study indicated that the adolescents aged 13–18 with standard body mass more often had hyperkyphosis and funnel (sunken) chest than obese children.

In this age group, a considerable decline in interest in sports activities is observed; concurrently, obese teenagers tend to be excused from physical education classes at school. Obese students habitually spend their leisure time on watching TV and playing computer games than their slimmer friends [8, 33].

At present, more and more attention is drawn to the deficiency of vitamin D in children and adolescents, which might constitute a factor conducive to the incidence of incorrect body posture [16, 29].

Excessive body mass predisposes not only to a higher incidence of incorrect body posture in children and adolescents, but it can also have negative impact on the whole metabolism, including the bone system [15–17, 26, 29, 41]. Considering the fact that the prevalence of these disorders among school children is high, one should stress the importance of preventive programs [34, 35, 42, 46]. Apart from increasing the level of physical activities and promoting healthy lifestyles, another key preventive action should be aimed at making the children adopt the habit of maintaining proper posture. The joint prevalence of incorrect posture and obesity among children calls for urgent preventive activities which would incorporate not only the factors related to excessive body mass but also age. Due to the fact that in the developmental period, a large number of cases with postural errors of functional nature are observed, the corrective gymnastic exercises should take into consideration the age of the studied population.

## Conclusions

The obesity was associated with incorrect body posture for both gender to 7–12 years of age, and the most common postural deviations in obese children and adolescents were valgus knees and flat feet. Overweight and obesity in children and adolescents, predisposing to higher occurrence of some types of postural errors, call for prevention programs addressing both health problems.

## Abbreviations

BMI: Body mass index
CI: Confidence interval
OR: Odds ratio
